# Primary care physicians’ narratives on COVID‐19 responses in Japan: Professional roles evoked under a pandemic

**DOI:** 10.1002/jgf2.452

**Published:** 2021-05-14

**Authors:** Junji Haruta, Sachiko Horiguchi, Junichiro Miyachi, Junko Teruyama, Shuhei Kimura, Junko Iida, Sachiko Ozone, Ryohei Goto, Makoto Kaneko, Yusuke Hama

**Affiliations:** ^1^ Medical Education Center School of Medicine Keio University Tokyo Japan; ^2^ Department of Primary Care and Medical Education Faculty of Medicine University of Tsukuba Tsukuba Japan; ^3^ Undergraduate Program Temple University Japan Tokyo Japan; ^4^ Azai‐higashi Clinic Hokkaido Centre for Family Medicine Nagahama Japan; ^5^ Center for Medical Education Graduate School of Medicine Nagoya University Nagoya Japan; ^6^ Faculty of Library, Information and Media Science University of Tsukuba Tsukuba Japan; ^7^ Faculty of Humanities and Social Sciences University of Tsukuba Tsukuba Japan; ^8^ Faculty of Health and Welfare Kawasaki University of Medical Welfare Kurashiki Japan; ^9^ Department of General Medicine and Primary Care Faculty of Medicine University of Tsukuba Tsukuba Japan; ^10^ Department of Health Data Science, Graduate School of Data Science Yokohama City University Yokohama Japan; ^11^ Tokyo Junior College of Transportation Tokyo Japan

**Keywords:** adaptive performance, contextual performance, COVID‐19, infectious disease, Japan, primary care, qualitative research

## Abstract

**Background:**

Within the vague system of primary care and COVID‐19 infection control in Japan, we explored how primary care (PC) physicians exhibited adaptive performance in their institutions and communities to cope with the COVID‐19 pandemic from January to May 2020.

**Methods:**

Narrative analysis conducted by a team of medical professionals and anthropologists. We purposefully selected 10 PC physicians in community‐based hospitals and clinics and conducted a total of 17 individual and group interviews. The verbatim transcript data were analyzed using the conceptual framework of adaptive performance.

**Results:**

We identified three “phases” of the time period (January–May 2020). In Phase 1, PC physicians initially perceived the disease as a problem unrelated to them. In Phase 2, the Diamond Princess outbreak triggered adaptive performance of the physicians, who began to deal with medical issues related to COVID‐19 by using social networking services and applying the collected information to their organization and/or communities. Following this, in Phase 3, the PC physicians’ adaptive performance in their own communities and institutions emerged in the face of the pandemic. Reflecting their sensitivity to local context, the PC physicians were seen to exhibit adaptive performance through dealing with context‐dependent problems and relationships.

**Conclusions:**

PC physicians exhibited adaptive performance in the course of coping with the realities of COVID‐19 in shifting phases and in differing localities in the early stages of the pandemic. The trajectories of adaptive performance in later stages of the pandemic remain to be seen.

## 1 INTRODUCTION

Across the globe, the COVID‐19 (coronavirus disease 2019) pandemic has transformed the lives of medical professionals. While experts have debated various factors for Japan's “success” in holding back “the first wave” in March–May 2020 (Table 1)^1^, ^2^, ^3^, ^4^ despite its lenient restrictions,^5^ data‐based analysis is scarce.

**TABLE 1:** Timeline of the “first wave” in Japan and around the world^1^, ^2^

Phase	Date	Event
Phase 1	2019/12/30	Li Wenliang posts an examination report and CT scan image on WeChat of a patient afflicted with unexplained pneumonia at Wuhan Central Hospital, China
	2019/12/31	First report of pneumonia of unknown origin submitted to World Health Organization (WHO)
	2020/1/7	A new coronavirus identified as the cause of unexplained pneumonia
	2020/1/9	First death from pneumonia caused by new coronavirus infection reported
	2020/1/16	Japan's first new coronavirus case reported
	2020/1/20	The Diamond Princess cruise ship, scheduled to return to port on 2/4, departs from Yokohama Port
	2020/1/23	Wuhan City enters lockdown
	2020/1/24	Chinese New Year holidays start (initially until 1/30 but later extended to 2/2)
Phase 2	2020/2/1	New coronavirus infection confirmed in a passenger who disembarked the Diamond Princess cruise ship in Hong Kong from the Diamond Princess cruise ship
	2020/2/5	The Diamond Princess cruise ship begins 14‐day quarantine off Yokohama in Kanagawa Prefecture, as ordered by the Japanese government
	2020/2/11	WHO names the disease caused by the new coronavirus infection “COVID‐19”
	2020/2/13	First COVID‐19 coronavirus death in Japan confirmed
	2020/2/16	Tokyo Metropolitan Government announces that about 100 people were considered to be in close contact at a New Year's party on a houseboat on 1/18
Phase 3	2020/2/21	Total number of COVID‐19 infections in Japan exceeds 100
	2020/2/27	Japanese government calls for the temporary closure of all elementary and secondary schools. Second COVID‐19 death confirmed in Japan
	2020/2/28	State of emergency declared in Hokkaido (mainly Sapporo)
	2020/3/11	WHO declares COVID‐19 outbreak a pandemic
	2020/3/21	Total number of COVID‐19 infections in Japan exceeds 1000
	2020/3/24	Japanese government announces the postponement of the Tokyo 2020 Olympic and Paralympic Games
	2020/3/25	Over 40 COVID‐19 infections in Tokyo were reported. Tokyo Governor declares Tokyo to be in a phase where an explosion of infections (“*overshoot*”) could occur and requests Tokyo residents to stay indoors. Ken Shimura, a famous Japanese comedian, reported to be afflicted with COVID‐19.
	2020/3/28	Hospital‐acquired infections were reported at an elderly welfare facility in Chiba and a hospital in Tokyo, Japan. Over 40 COVID‐19 infections in Tokyo
	2020/3/29	Ken Shimura's death from COVID‐19 pneumonia reported. Total number of COVID‐19 infections in Japan exceeds 2000
	2020/4/1	Japan's prime minister announces distribution of cloth masks and restrictions on departure and travel
	2020/4/3	Total number of COVID‐19 infections in Japan exceeds 3000
	2020/4/7	State of emergency declared in Japan for 7 prefectures, including Tokyo, until 5/6 on a voluntary basis
	2020/4/18	Total number of COVID‐19 infections in Japan exceeds 10,000
	2020/4/22	Total number of COVID‐19 deaths in Japan exceeds 200
	2020/5/2	Total number of COVID‐19 deaths in Japan exceeds 500
	2020/5/3	Total number of COVID‐19 infections in Japan exceeds 15,000
	2020/5/4	State of emergency declaration in Japan extended to 5/31
	2020/5/14	State of emergency declaration lifted in 39 prefectures (of a total of 47 prefectures) due to declining number of COVID‐19
	2020/5/21	State of emergency declaration lifted in 3 prefectures in the Kansai region (Osaka, Kyoto, Hyogo). State of emergency declaration lifted in 39 prefectures (of a total of 47 prefectures) due to declining number of COVID‐19 infections
	2020/5/24	Over 200 infections per million in metropolitan Tokyo and Osaka metropolitan areas
	2020/5/25	State of emergency declaration lifted in 5 remaining prefectures in the Kanto region and Hokkaido (Tokyo, Kanagawa, Saitama, Chiba, and Hokkaido)

Primary care (PC) physicians on the front line of community efforts to cope with infectious diseases should intervene quickly to the pandemic in principle.^6^ PC physicians around the world are struggling to cope with COVID‐19,^7^, ^8^, ^9^, ^10^ but how are PC physicians in Japan coping with the pandemic? We should begin by highlighting the structural context they are situated in. The boundaries between primary/secondary care and clinic/hospital care in Japan are more ambiguous than elsewhere, and the roles of PC physicians working under a universal health insurance and free access healthcare system are not clearly demarcated.^11^ Primary care is practiced not only by PC physicians based in clinics but also by hospital physicians whose main role is in secondary care.^6^

Moreover, Japan lacks a centralized command system of government and/or medical experts for COVID‐19 responses. Japan does not appoint a Government Chief Scientific Adviser as in the U.K., nor does it house an equivalent to the CDC (Centers for Disease Control and Prevention) in the United States or the State Council in China,^7^, ^12^ which coordinate joint multi‐sectoral responses to pandemics and provide resources for disease control. In February–March—the initial stages of the pandemic—the Japanese government asked public health centers (PHCs) to play a gate‐keeping role for COVID‐19, but the PHCs were overwhelmed by increasing demands.^13^ Consequently, PC physicians were tasked with triaging patients with suspected COVID‐19 and were forced to facilitate early detection in the community with limited medical evidence and a lack of resources.

To outline the experiences of PC physicians coping with the ever‐changing pandemic in these challenging circumstances, we shed light on their adaptive performance. Koopsman et al define adaptive performance as “adapting to changes in a work system or work roles,” which can include “adjusting goals and plans according to situation, generating new innovative ideas, learning new tasks and technologies, understanding other groups or cultures, being flexible and open‐minded towards others, showing resilience, quick analysis, remaining calm, and acting appropriately.”^14^ Exploring the PC physicians’ adaptive performance in response to COVID‐19, in a structural context where their roles are ill‐defined—both socially and institutionally and in terms of infection control—will enhance our understanding of PC physicians’ roles in uncertainty. In the following, we illustrate the trajectories of their adaptive performance under the COVID‐19 pandemic.

## 2 METHODS

### 2.1 Study design

Our study aims to explore PC physicians’ adaptive performance in response to COVID‐19. To capture ways in which the physicians struggled to adapt their practices in a timely manner, we adopted social constructionism,^15^ which focuses on how human behaviors contribute to constructions of perceived social reality and ways in which knowledge and meanings are predicated on social context, as a paradigm for our research. Based on this paradigm, we used “narrative inquiry,” employed in examinations of infectious disease pandemics,^15^, ^16^, ^17^ as a methodological framework.^18^ Narrative inquiry is a research method where commonalities observed in story‐telling are interpreted and analyzed.^19^ Typologies of narratives are inductively created, conceptually organized by thematic categories, and illustrated by exemplar narratives or vignettes.^18^, ^20^ The qualitative approach we have undertaken, supported by the anthropologists in our team, has allowed us to capture complex perceptions and emotions of the physicians that are at times ambivalent and hence difficult to grasp in quantitative studies and to refine our interpretations through multifaceted examination. This study has been conducted since March 2020, and here, we focus on the narratives on their experiences from January to May, during which there was a high degree of uncertainty and lack of knowledge about COVID‐19 and its future course.

### 2.2 Data collection

**FIGURE 1:**
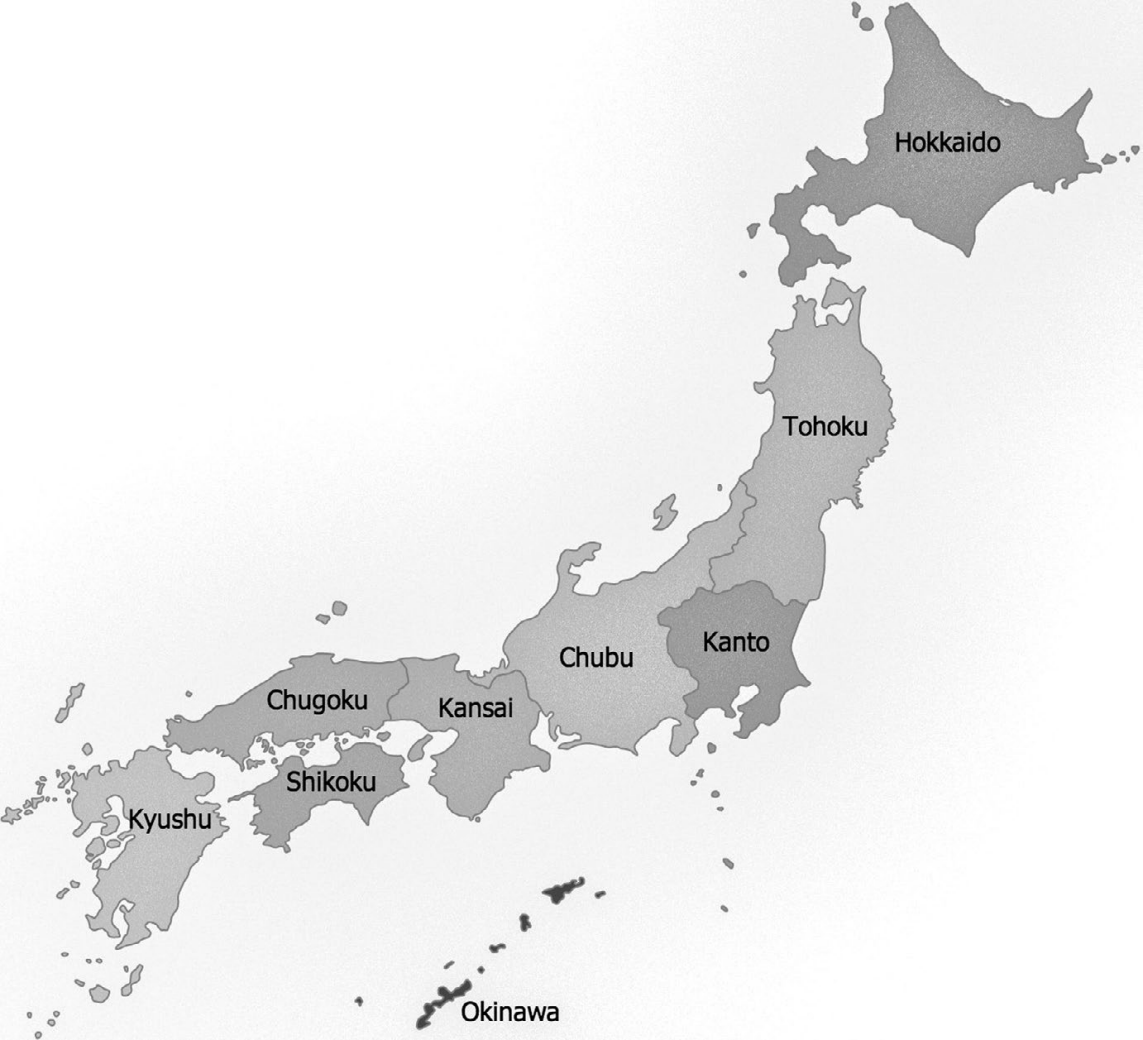
The locations of the regions in Japan

The researchers—all native Japanese speakers in our thirties and forties—used our personal networks to select 10 JPCA (Japanese Primary Care Association)‐certified PC physicians through purposive sampling which assured regional diversity and balance in the number of physicians in hospitals and clinics. After the physicians gave their consent to participate in the study through e‐mail, our research team conducted online interviews using Zoom, the videoconference application, from March (Tables 2 and 3; Figure 1). We asked interviewees to tell us about when they first learned about COVID‐19 and what they have experienced and felt in their everyday practices in response to the pandemic over time. During the interviews, we presented a table of events related to COVID‐19 similar to Table 1 to help our interviewees recollect their memories. By the end of June 2020, we had conducted a total of 17 individual and pair interviews with the 10 participants. JH conducted our first Zoom interview with Dr. A, followed by another with Dr. F by JH and JT. The recorded videos of these interviews were shared in the team to acquaint ourselves with interview procedures. A team of 1–3 medical professionals and 1–2 anthropologists then teamed up to interview one physician, with one medical professional serving as the main interviewer, considering the closeness in their professional context to the interviewees. The sub‐interviewers gave additional questions and took detailed notes on the narratives and their ethnographic observations. After a round of individual interviews (except for Dr. A, with whom we had two individual interviews before the pair interviews), to facilitate meaningful interactions among participants, we created five pairs of the interviewees and assigned teams of two medical professionals and two anthropologists to interview each pair. This has helped us build rapport with the physicians and observe the changes in their practices. Interviews were initiated at different time periods depending on the interviewee, and the frequency of interviews also varied slightly. This was partly because an issue with gender bias was addressed in our team in late April and two female participants were added to the study, after noticing that the first batch of interviewees were all male. We have considered requesting more interviews with the spread of COVID‐19 all over Japan, but decided not to do so due to concerns about sustainability. The study is ongoing, but we limit the analysis in this article to the events up to May 2020—often dubbed the end of Japan's “first wave” of COVID‐19—and narratives collected until June 2020. Verbatim transcripts were created from recorded data and were analyzed together with the sub‐interviewers’ notes. The entire process of interviewing and analyses was conducted in Japanese, and the narrative data were translated into English for this publication.

**TABLE 2:** Backgrounds of study participants

	Gender	Workplace	Institution	Roles associated with COVID‐19	Work context and training
A	M	Metropolitan areas: Kanto region	Hospital & clinic	Screening, specimen collection, and fever outpatient services in a hospital and a clinic; follow‐up of mild COVID‐19 patients in a hospital	Community hospital and a small private clinic (with two doctors) as a trainee resident of family physicians in a metropolitan area; trained in the field of infectious diseases
B	M	Metropolitan areas: Kansai region	Clinic	Screening and maintenance of a fever outpatient clinic as a director	Small clinic (with one doctor) in a metropolitan area
C	M	Metropolitan areas: Kanto region	Clinic	Screening and maintenance of a fever outpatient clinic; management of staff as a director; specimen collection in local PCR testing center	Private clinic that runs a group practice near the city where the Diamond Princess cruise ship arrived
D	M	Regional cities & suburban areas: Hokkaido	Clinic	Screening and maintenance of a fever outpatient clinic; management of staff as a director	Public clinic (with some doctors) in a town of about 4000 people
E	F	Regional cities & suburban areas: Chubu region	Clinic	Screening and maintenance of a fever outpatient clinic; management of staff as a director	Public clinic (with some doctors)
F	M	Hospitals serving COVID‐19 patients: Kanto region	Hospital	Screening and maintenance of fever outpatient services; treatment of mild or moderate COVID‐19 patients; management of staff as a director of a department	Department of internal medicine at a publicly run designated hospital for infectious diseases
G	M	Regional cities & suburban areas: Chugoku region	Clinic	Screening and maintenance of a fever outpatient clinic; management of staff as a director	Private clinic that runs a group practice
H	M	Hospitals serving COVID‐19 patients: Kanto region	Hospital	Screening, specimen collection, and maintenance of fever outpatient services and treatment of mild or moderate COVID‐19 patients in a hospital	Department of general medicine at a public hospital (not designated as a hospital for infectious diseases)
I	M	Regional cities & suburban areas: Kyushu region	Hospital & clinic	Screening in some clinics	University hospitals and clinics throughout the prefecture, including those on remote islands
J	F	Regional cities & suburban areas: Chubu region	Clinic	Screening, specimen collection, and maintenance of a fever outpatient clinic; management of staff as a director	Private clinic that runs a group practice as a board‐certified infectious disease specialist

Abbreviations: F, Female; M, Male.

**TABLE 3:** Interview dates, interviewees and interviewers, and duration of the interviews

Date	Interviewee		Interviewer					Duration of the interview
2020/3/24	A		JH^a^					66 min
2020/3/26	F		JH^a^	JT				62 min
2020/3/27	I		JH^a^	SO	SH			105 min
2020/4/2	G		JH^a^	YH				90 min
2020/4/3	H		JH^a^	JM	SK			79 min
20204/7	D		JM^a^	JH	JI			106 min
2020/4/14	A		JH^a^	JI				54 min
2020/4/16	C		JH^a^	RG	SH			61 min
2020/4/23	G	D	JH^a^	MK	SO	JI		88 min
2020/4/25	F	H	JH^a^	RG	JT	SH		88 min
2020/5/3	E		JH^a^	RG	SK			87 min
2020/5/12	B		JM^a^	MK	YH	SH	JI	121 min
2020/5/15	J		MK^a^	JM	SH	YH		82 min
2020/5/19	A		JH^a^					54 min
2020/6/6	I	B	SO^a^	JM	SH	JT		96 min
2020/6/11	A	E	JH^a^	RG	SK	JI		80 min
2020/6/17	F	H	JH^a^	RG	JT	SH		83 min

^a^ Main interviewer.

### 2.3 Researchers’ backgrounds

Our research team is comprised of four primary care physicians, one physical therapist, and five anthropologists, all of whom are Japanese. Among the medical professionals, JH received training in qualitative research as part of a PhD program, JM obtained a Master's degree in medical anthropology, and SO, RG, and MK received qualitative research training after obtaining their PhD degrees. The anthropologists hold doctorates in anthropology and have about 10–20 years’ experience in their fields: SK and SH specialize in socio‐cultural anthropology while JI, JT, and YH are medical anthropologists. The research team members had collaborated in different interdisciplinary projects prior to this research.

### 2.4 Analysis

In this study, we found patterns in the interviewees’ narratives, while taking account of the variations in the number of infections by region, type of institution they work for, and time period. We then identified and streamlined the adaptive performances revealed in the narratives. The texts were analyzed primarily by JH, who identified key themes that emerged consistently across a series of narratives. The research team members then reviewed the data including its appropriateness in sampling and critically examined these themes to maintain the robustness of the data and analyses.^18^, ^19^ Our discussions with the anthropologists on the team who emphasize the contexts in which our interviewees are situated has encouraged us to be reflexive throughout the research process.

### 2.5 Ethical consideration

The JPCA Ethics Committee approved this research project. All study participants provided informed consent prior to participation. To protect the anonymity of the interviewees, quotes in this paper are identified by randomly assigned alphabet codes rather than participants’ names or initials.

## 3 RESULTS

A total of 17 interviews were conducted with 10 PC physicians in their thirties and forties (Tables 2 and 3).

### 3.1 Findings

We divided the period between January and May 2020 into three phases to analyze the PC physicians’ shifting adaptive performance, based upon the transitions recollected in their narratives and the changing situations in Japan surrounding COVID‐19: Phase 1, seeing the epidemic as an othered problem; Phase 2, sensing the pandemic reality approaching their communities; and Phase 3, facing the pandemic as an everyday reality in their communities. Due to regional variations in the timing of the pandemic outbreaks, each phase was experienced in different time periods depending on the region where the physicians worked. Readers can see Table 4 for more information on the narratives.

**TABLE 4:** Narratives of PC physicians under study

Phase	Trajectories of adaptive performance	Quotes
Phase 1: Seeing the epidemic as an othered problem	Mostly recognizing no need for adaptive performance associated with COVID‐19	Dr. A: “At first, I really didn't have any information [about the new coronavirus] nor did I know anything about it yet. … I felt like it was occurring in a completely distant country.” Dr. B: “There's a lot of inbound traffic around here. So, when we heard in mid‐January that there's an infectious disease in China, our staff as well as our patients who regularly come to the clinic started saying that things are going to get bad around here as well. Even though I heard these people talking about it, it seemed distant to me. I didn't realize how bad it was, and I thought it was mostly just a minor illness.” Dr. D: “I thought that containment of COVID‐19 would take place there [in China]. For example, there have been deaths in China from the occasional outbreak of bird flu, H5N1 and things like that, and I thought it would be contained in the same way.” Dr. E: “I went to Nagoya with my family in January. There were so many Chinese people all over Nagoya, and you could hear almost only Chinese in the subways and hotels. … And when we got back, I was nervous that the COVID‐19 epidemic was going to be terrible. … at that time … there was a feeling somewhere that it wasn't going to be okay. … But at that point, it was still something distanced from us.” Dr. J: “[I think I first heard about the coronavirus] around January 20‐something, … around when the first case surfaced [in Japan]…. I learned that something similar [to MERS and SARS] had surfaced and that it was spreading rapidly, so I was really hoping that it wouldn't be like MERS or SARS.”
Phase 2: Seeing the pandemic reality approaching their communities	Exhibiting adaptive performance and undertaking new tasks to deal with COVID‐19 with little time to spare	Dr. C: “I felt that I had to fulfill my responsibility as a doctor to the people aboard the Diamond Princess. Considering the situation in Japan, even if I wanted to conduct PCR testing, I didn't think I would be able to do so. … [Then) the medical association asked me to do it on [Friday] February 14th, but I got a call on Thursday night, and they said you're a member of the first team.” Dr. F.: “The Diamond Princess had the biggest [impact]. I heard that the infection had spread quite a bit on the cruise ship. A DMAT (Disaster Medical Assistance Team) at our hospital had to go on the cruise ship. We sent one team from our hospital. … They said that when they disembark from the ship, they might have to send the patients to hospitals located across a broad geographical area, so I guessed that we would have to accept [COVID‐19 patients] in our hospital. I also contacted the [local] health center and told them that we had two beds for accepting patients. This was probably in early to mid‐February.” Dr. H: “Our hospital started to prepare for COVID‐19 when we heard that there was a massive outbreak on the Diamond Princess and that the designated infectious diseases hospitals didn't seem to be able to handle it alone. I remember it was around early to mid‐February that a task force for the infection control of COVID‐19 was set up to discuss how to handle the outbreak, who would examine the patients, and how to deal with the situation.”
	Exhibiting adaptive performance through discerning credible information on COVID‐19 and preparing for future outbreaks	Dr. A: “There were so many different opinions swirling around on Twitter and other places. There were a lot of people blaming others without understanding the reason. There were a lot of hoaxes going around. After I realized that, I had a hard time looking at Twitter, so I stopped tweeting.” Dr. B: “I don't usually read Facebook much, but with regard to COVID‐19, I always turned to it. I'm grateful that a trusted infectious disease specialist, who I know in person, posted trustworthy information.” Dr. D: “I referred to information sent out by doctors who curated the scattered information and who knew a lot about what was going on inside the Diamond Princess [cruise ship]. They posted important information on Facebook, and if I followed their posted links, I would be taken to important texts from the Ministry of Health, Labor and Welfare. So, I always followed the postings on Facebook by those who selected and shared valuable information.” Dr. F: “The information came from a thread on Facebook on how medical professionals coping with COVID‐19 dealt with the condition. We would look at it and ask questions from time to time, and share the information in our hospital. … I checked with each doctor to make sure that each piece of information was applicable in our hospital setting. We drafted the policies at our hospital based on this information.” Dr. G: “Through Facebook, … I felt that something strange was going on when competent people … started arguing over each other's comment. I feel like it was something that was too soon to be considered a crisis. I am not sure, but I think social networking has had a big impact on me, and if I hadn't been on Facebook, I would have felt more like the problem was not my concern.”
Phase 3: Facing the pandemic as an everyday reality in their communities	(1) Hospitals serving COVID‐19 patients: Exhibiting swift adaptive performance through acquiring knowledge, solving problems creatively, handling emergencies and work stress, dealing with uncertain and unpredictable work situations, learning work tasks and procedures, and demonstrating interpersonal adaptability	Dr. F: “During the weekend from March 14th to 15th, I created a manual (=guidelines for infection control in the hospital). I shared it with my colleagues on March 16th, but it alone won't work. … So I collaborated with ICN (nurses specializing in infectious diseases) … and we created ‘action cards’, so that each department can quickly move and check [their] movements on a sheet of paper.” “I think that the movement of people in March and April is dangerous for this infection, and there is a possibility that many people will be infected with COVID‐19.” (Note: In Japan, the school year as well as the fiscal year for many private corporations starts in April and ends in March. Therefore, this is a season when many people relocate, change jobs, and mingle with new acquaintances.) (In discussing the institutional strategies of personnel management in the hospital) “[We made sure that] COVID‐19 patients are attended by different physicians. It's become a Russian roulette within the internal medicine team. This is a good way to engage everyone and to encourage everyone's involvement in COVID‐19. On the other hand, it also exposes more people to the risk of being infected. … There are also differences in the awareness level between staff in the wards who see COVID‐19 patients and those who don't.” Dr. H: “We set up a booth in the emergency room for febrile patients and set up a waiting area near it. We struggled to figure out how to divide up the space so that there would be no intersection with other patients. … When I asked for a CT scan of a suspected COVID‐19 patient, our staff answered ‘can I do it in the evening because there would be no other patients then?’ I was like, no, no, no, I'm telling you there's a patient here right now…” “We had to divide our staff into days of the week to see people with fever in a container – a prefabricated hut – outside the hospital, once a week. The respiratory specialist took on that role with, well, what I think is a sense of mission. It's a respiratory issue so they'd have to be involved. The PC physicians also took on that role with a sense of mission.” (With a hint of fatigue on his face) “When a patient is admitted to the hospital, there is a lot of nervousness about whether the patient's condition has worsened and whether he or she needs to be transported to a university hospital. We have to wear personal protective equipment, manage multiple people on the ward, and perform PCR testing for negative confirmation before discharging the patient from the hospital. The number of not only young but also elderly patients is increasing, so there are more and more people who need help. We need to ask other departments’ doctors to handle the outpatient services. I've asked the hospital to send someone to the team. Recently, my practice has been limited to only COVID‐19 or suspected patients. I rarely conduct physical examinations or anything else to avoid contact with these patients.” “When I asked one of the doctors on night duty to take the call from the health center and record the results of the PCR testing, he said, ‘Is that my job?'”
	(2) Metropolitan areas: Exhibiting adaptive performance through adjusting their priorities while dealing with uncertain and unpredictable work situations, learning work tasks and procedures, and demonstrating interpersonal adaptability	Dr. A: “I'm seriously thinking of starting a full‐fledged version of telemedicine practice. We're talking about which company is better for online practice and when the system will actually be ready. We're discussing the issues of how to cope with patients who are known to be positive and are staying at home.” “We're told that we need to reduce the amount of time we spend with patients, so I don't have the opportunity to talk to them for so long. Patients themselves don't want to see each other for long periods of time because many of them really just want to receive medications." Dr. B: “Towards the end of January, we talked about what to do when a patient is suspected of having COVID‐19. The procedural manual was completed in the clinic by February 14th. … Since that time, we've tried to separate the places completely and properly, and if people had cold symptoms in the waiting room, we asked them to go upstairs. We kept the first floor for seeing regular patients. … It wasn't until March 10th that the entrance to the symptomatic patients was completely separated. [Around that time,] I started wearing goggles, a mask, gloves and an apron when seeing these patients. We started opening windows.”
	(3) Regional cities and suburban areas: Exhibiting adaptive performance within and for their institutions and their communities through handling work stress and making the most of their interpersonal adaptability	Dr. E: “We think of ourselves as physicians in the community, and I thought it was very important to send out information to residents we provide medical services to as it relates to this community. … So, from the end of February, we began to hold study meetings, step by step, to discuss things like social distancing and time separation. We talked about it at every study meeting. We delivered information to residents in the local community about how to prevent COVID‐19. We thought about what we should do in our daily lives, rather than just ordering people or telling them to do this or that.” Dr. G: “During a morning meeting on March 9th, I told our staff in the clinic that the outbreak had also occurred in our prefecture. At that time, I told them that it would be a six‐month to two‐year battle, as a nationwide epidemic would be unavoidable. … I think our staff were worried that the elderly and those with chronic illnesses in the clinic might get infected with COVID‐19, especially since they would have to be hospitalized. I told the staff members that it's important to protect our patients and ourselves, and assume that it [=COVID‐19] will slowly see outbreaks in our community.” Dr. J: “I think our staff members understand that they have to handle the situation properly. So when I give them instructions and explain the rationale behind them, they follow the instructions without hesitation. No one wants to quit ‐ they are all very cooperative. … Normally, people make appointments online or by phone, but we get calls from people in the community who are hesitant about coming to the clinic. The staff would then explain over the phone that we are dividing up the time slots for patients so they don't have to worry. During the evening review, we would hear that the patients were very happy when we explained to them in this way, or that they came to the clinic feeling safe and comfortable. Hearing these stories, we all praise each other for handling the situation properly.”

#### 3.1.1 Phase 1: Seeing the epidemic as an othered problem

In the initial stages of the global outbreak of COVID‐19—January 2020—despite the geographical proximity of the outbreak in neighboring China, the PC physicians perceived COVID‐19 as a problem that does not directly concern them. They also had a hopeful assumption that the outbreak would be contained. Dr. A confided that with a lack of information, he “felt like it was occurring in a completely distant country.” Dr. B similarly said, “It seemed distant to me. I didn't realize how bad it was, and I thought it was mostly just a minor illness.” At this point, except for Dr. J, who specializes in infectious diseases (see Table 4), the physicians did not recognize a need for adaptive performance associated with COVID‐19, since it was considered as an othered problem, and their job performance seemed unchanged from their usual routine.

#### 3.1.2 Phase 2: Sensing the pandemic reality approaching their communities

In Phase 2, the PC physicians increasingly came to a realization that COVID‐19 was to be a disease they would have to cope with. The outbreak in February on the Diamond Princess cruise ship (Table 1) forced some physicians to exhibit adaptive performance and undertake new tasks to deal with COVID‐19 with little time to spare. Dr. F, working in the general practice department of a designated infectious disease hospital, encountered a situation, which required responding to a local emergency. He noted how contacted the [regional] PHCs to “tell them that we [=they] had two beds for accepting patients.”

During this period, due to a realization that COVID‐19 was looming in time and space, the physicians started gathering information on COVID‐19 using social networking services, especially Facebook, which functioned as a tool for collecting, reporting, and spreading information. Dr. D “referred to the information shared [on Facebook] by doctors who curated the scattered information and knew a lot about what was going on inside the Diamond Princess [cruise ship].” From this phase, except for those who dealt with the Diamond Princess outbreak and those working near Yokohama, the physicians gradually started demonstrating their adaptive performance through discerning credible information on COVID‐19 and preparing for future outbreaks.

#### 3.1.3 Phase 3: Facing the pandemic as an everyday reality in their communities

Since the end of February, the PC physicians were forced to exhibit adaptive performance in response to the shifting realities of COVID‐19 in their localities. Here, we grouped the physicians exposed to different everyday realities in the following manner: those working in (1) hospitals serving COVID‐19 patients, (2) metropolitan areas, and (3) regional cities and suburban areas.

##### Hospitals serving COVID‐19 patients

The PC physicians working in hospitals serving COVID‐19 patients were forced to not only urgently face the demands of coping with medical issues related to COVID‐19, but also to immediately figure out how to deliver information on dealing with suspected COVID‐19 patients and mapping institutional flows to cope with COVID‐19. Dr. F mentioned creating a manual for dealing with infected or suspected cases and sharing it with his colleagues on March 16th, which did not work. They then created “action cards,” which “mapped out key procedures on a sheet of paper, so that each department could move quickly.”

In the beginning of April, as the number of encounters with COVID‐19 patients increased day by day, the duties of dealing with COVID‐19 patients became embedded in the physicians’ daily routines, which caused physical and mental fatigue. Even in their busy routines, the physicians became aware of the significance of their context, such as in identifying problems with how information was shared with hospital staff, and quickly making the accommodations required to remedy these issues. Dr. H said with a hint of fatigue on his face, “We set up a booth in the emergency room for febrile patients and set up a waiting area near it. We struggled to figure out how to divide up the space so that there would be no intersection with other patients.”

The PC physicians working in hospitals were found to display their swift adaptive performance in various dimensions. They solved problems creatively while handling emergencies and work stress, dealt with uncertain and unpredictable work situations, learnt work tasks and procedures, and demonstrated interpersonal adaptability.

##### Metropolitan areas

The PC physicians working in medical institutions in large cities (Tokyo, Osaka, etc.) had to begin following the specific flows of COVID‐19 responses early on in February, because they were already starting to face the realities of the COVID‐19 outbreaks in their immediate localities (Tables 1 and 2, 2/16, 2/21). In Dr. B’s case, “The procedural manual was completed in the clinic by February 14th. It wasn't until March 10th that the entrance to the symptomatic patients’ clinic was completely separated from other patients.” He started “wearing goggles, a mask, gloves and an apron when seeing patients” and “opening windows” around that time. Others working in metropolitan clinics and hospitals serving many patients in limited space began to consider introducing new systems, such as a “telemedicine system” as in the case of Dr. A., to prevent the clustering of patients in their facilities. Dr. A also visited PHCs in March to interview health workers about their COVID‐19‐related tasks COVID‐19 and what his clinic could do. The PC physicians’ work therefore involved not only serving their patients and preventing outbreaks within their institutions, but also reaching out to their communities and being sensitive to their needs as guardians of their local communities.

In response to the increasing number of infections and the state of emergency declaration (Table 1, April) in April, the examinations and treatments of outpatient fever cases were incorporated into regular clinical routines for the physicians in these regions. Some PC physicians started avoiding extended medical interviews and physical examinations to minimize their contact with their patients. Dr. A lamented having to focus on “laboratory tests, and not on the physical examination” and noted that he “didn't want to do this.” He was asked to “reduce the amount of time I [=he] spent with patients, so I [=he] didn't have the opportunity to talk to them for so long. … Many of them really just want to receive medications.” In the course of being forced to quickly adapt to a new clinical style, the physicians were faced with dilemmas of foregoing long medical interviews or physical examinations, which practice lies at the core of primary care.

The PC physicians in metropolitan areas therefore exhibited adaptive performance, through adjusting their priorities while dealing with uncertain and unpredictable work situations, learning work tasks and procedures, and demonstrating interpersonal adaptability.

##### Regional cities and suburban areas

In regional cities or suburban areas, learning about the first COVID‐19 case in the physicians’ neighborhood changed their perceptions. Although the opportunities to exhibit adaptive performance to work directly with COVID‐19 and suspected patients were limited, the predictability of the COVID‐19 outbreak in their immediate context enhanced their awareness of their adaptive performance, such as collecting and sharing information on COVID‐19 about how to protect individuals from infections or to prevent the transmissions, and learning about the pandemic together with their staff. Dr. G works at a clinic in a regional city wherein the first COVID‐19 case surfaced in early March. After explaining that his clinic houses inpatient facilities and his staff includes elderly care professionals, he “told the staff members that it's important to protect our [=their] patients and ourselves [=themselves], and assume that COVID‐19 outbreaks will slowly surface in our community.”

The PC physicians working in regional cities or suburban areas made efforts to relieve the staff's anxiety level in preparing for potential outbreaks in their local context and to summarize and share specialized knowledge on COVID‐19 with the healthcare staff in their institutions and local communities. They therefore demonstrated adaptive performance within and for their institutions and communities through handling work stress and making the most of their interpersonal adaptability.

## 4 DISCUSSION

As we have seen, PC physicians exhibited adaptive performance in the course of coping with the realities of COVID‐19 in various phases and localities. Initially, the physicians did not necessarily recognize a need for adaptive performance associated with COVID‐19, but facing the pandemic, some physicians working in hospitals had to demonstrate their adaptive performance immediately. Others in metropolitan areas needed to adapt quickly to cope with local outbreaks, while those in regional cities gradually adapted to prepare for imminent outbreaks. The key factors of adaptive performance^14^, ^21^ were demonstrated in shifting phases and differing local contexts in coping with the realities of COVID‐19.

Although the PC physicians have been found to display adaptive performance, it is important to note that some experienced dilemmas in this process. The last 20 years have seen transitions in primary global health concerns from infectious diseases to noncommunicable diseases (cardiovascular diseases, cancers, diabetes, and chronic respiratory diseases).^22^ Particularly in Japan, PC physicians’ practices have focused on responding to the super‐aging society and a need for the prevention of capillary regression under disuse conditions.^6^, ^23^, ^24^ They typically spend a relatively long time to be a reliable contact for patients’ health concerns and directly address most healthcare needs at first contact.^25^ Partly due to this, Japanese PC physicians may find it difficult to respond immediately to a pandemic, as was observed in Phase 1.

We found, however, that enhanced communication with colleagues, patients, staff in PHCs, and citizens— a key in adaptive performance—and creating and maintaining a supportive environment have helped PC physicians cope with the high level of uncertainty under the pandemic. In an increasingly complex society, not only adaptive performance but also contextual performance has been seen as essential for effective job performance.^26^, ^27^, ^28^ Contextual performance is reflected in activities that support the organizational, social, and psychological environment, such as motivation, cooperation with and helping others, communication, interpersonal relations, and organizational commitment.^29^ It is evident that PC physicians were required to show contextual performance in order to achieve effective adaptive performance in response to the pandemic. The fact that they are trained to be sensitive to context may have positively impacted their adaptive performance.^30^ While strict requirements for adherence to practice guidelines in training and service design in the U.K. may limit the development and the implementation of individualized and contextualized care from GPs,^31^ the vagueness of PC physicians’ roles in Japan may have contributed to the physicians’ high contextual performance. These PC physicians’ adaptive performance could be keys in coping with the pandemic,^32^ and the trajectories of their adaptive performance in the early stages of the COVID‐19 outbreak may potentially be applicable to those medical professionals responding to the subsequent waves of the pandemic and beyond, although further investigation of the adaptive performances of these PC physicians in later stages of the pandemic is needed to critically examine this applicability. This study offers insights into the constantly changing pictures of the pandemic based on the physicians’ fresh memories, and the anthropologists’ involvement in the study has helped enhance our contextual insights.

There are, however, several limitations. This is a small‐scale study based on purposive sampling with a significant lack of variety in terms of age and gender, and a more macro‐level study are needed for a comprehensive analysis of PC physicians’ responses to COVID‐19. Using the researchers’ personal networks to seek interviewees may have not only led to this limitation but also affected our interactions and analyses. Not all Japanese PC physicians may interpret their roles as described above, and considering the increasingly severe pandemic situations in more recent months, our current observations on the effectiveness of their adaptive performance may be overly optimistic. A longitudinal investigation of the transitions of their adaptive performance is therefore needed.

## 5 CONCLUSION

Faced with a need to cope with the changing realities COVID‐19, PC physicians in Japan exhibited effective adaptive performance in shifting phases and differing contexts in the early stages of the pandemic. The trajectories of adaptive performance to be demonstrated by the PC physicians in later stages of the pandemic remain to be seen.

## CONFLICT OF INTEREST

The authors have stated explicitly that there are no conflicts of interest in connection with this article.
